# Atypical Intraparenchymal Meningioma with YAP1-MAML2 Fusion in a Young Adult Male: A Case Report and Mini Literature Review

**DOI:** 10.3390/ijms241612814

**Published:** 2023-08-15

**Authors:** Alisa Nobee, Mei Xu, Anjali Seth, Yuan Rong

**Affiliations:** 1 Department of Pathology and Laboratory Medicine, Temple University Hospital, Philadelphia, PA 19140, USA; 2 Department of Biomedical Sciences, Philadelphia College of Osteopathic Medicine, Philadelphia, PA 19131, USA

**Keywords:** atypical meningioma, intraparenchymal meningioma, YAP1-MAML2 fusion

## Abstract

Oncogenic Yes-associated protein (YAP) 1 fusions have been recently identified in several cases of meningioma mostly involving pediatric patients. The meningiomas harboring YAP1-MAML2, which is the most frequent fusion subtype, exhibit activated YAP1 signaling and share similarities with *NF2* (neurofibromatosis type 2 gene) mutant meningiomas. We reported a rare case of atypical intraparenchymal meningioma with YAP1-MAML2 fusion in a 20-year-old male. The patient presented with an episode of seizure without a medical history. MRI revealed a lesion in the right temporal lobe without extra-axial involvement. The radiological and morphological findings, however, were indistinctive from other intracranial diseases, e.g., vascular malformation and glioma. Immunohistochemical results confirmed the presence of abundant meningothelial cells in the tumor and indicated brain invasion, supporting the diagnosis of atypical intraparenchymal meningioma. Targeted RNA fusion analysis further identified a YAP1-MAML2 rearrangement in the tumor. Non-dural-based intraparenchymal meningiomas are uncommon, and the careful selection of specific tumor markers is crucial for an accurate diagnosis. Additionally, the detection of the fusion gene provides valuable insights into the oncogenic mechanism of meningioma.

## 1. Introduction

Meningiomas are the most common type of primary intracranial tumor, characterized by their slow growth and predominantly benign nature. They constitute approximately 37% of all central nervous system tumors and 53% of non-malignant cases. The majority of patients with meningioma are older adults, with a higher incidence in females [1,2]. Adolescents and young adult patients (AYAs), however, are exceedingly rare, only representing less than 1% [3]. The AYA meningioma presents distinct molecular and clinical characteristics, which are different from those of the older age group [4]. Previous studies have indicated that young patients with meningioma often have a risk of tumor predisposition syndrome [5,6]. Neurofibromatosis type 2 (NF2), now termed *NF2*-related schwannomatosis, is an autosomal dominant disorder that is believed to predispose individuals to the early development of meningioma [7,8,9]. NF2 is caused by mutations in the *NF2* gene, resulting in the loss of function of the tumor suppressor protein Merlin. Since Merlin negatively regulates YAP1 (yes-associated protein 1, a.k.a YAP) as an upstream activator of the Hippo signaling pathway, the deficiency of Merlin leads to the overexpression of YAP1 [10]. In recent years, several YAP1 fusions, including YAP1-MAML2, YAP1-PYGP1, and YAP1-LMO1, have been identified in a subset of pediatric meningiomas, with YAP1-MAML2 being the most common [6,11,12]. YAP1-MAML2 positive meningiomas have been reported to exhibit similarities to *NF2* mutant meningiomas, showing an elevated level of YAP1 signaling and a comparable response to a specific group of pharmaceutical reagents [13]. However, there have been very few case reports published on young adult patients with the YAP1-MAML2 fusion.

## 2. Case Presentation

**Figure 1:**
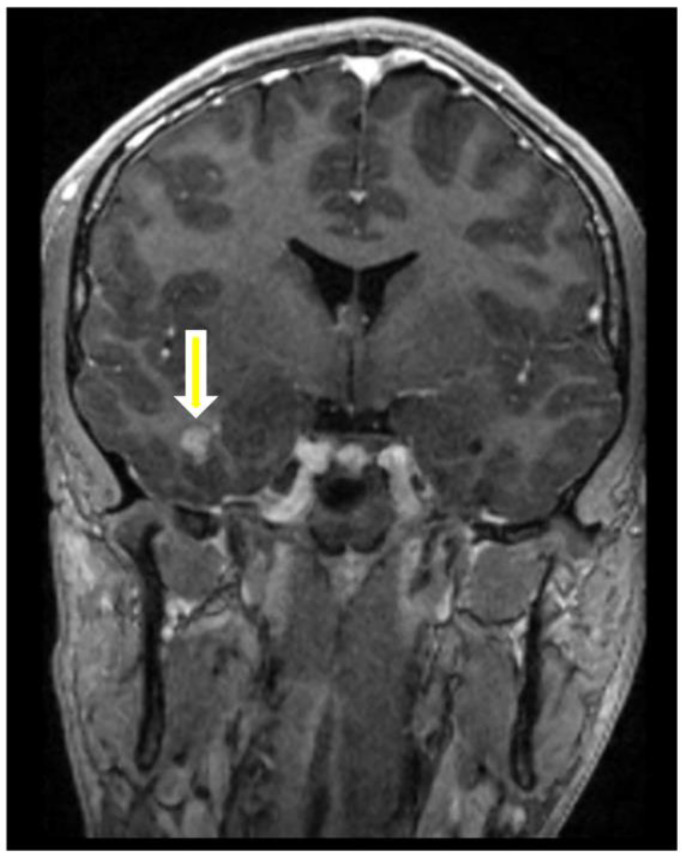
T1-weighted MRI post-contrast MRI revealing a lesion with heterogeneous enhancement in the right medial temporal lobe extending into the cortex (arrow).

**Figure 2:**
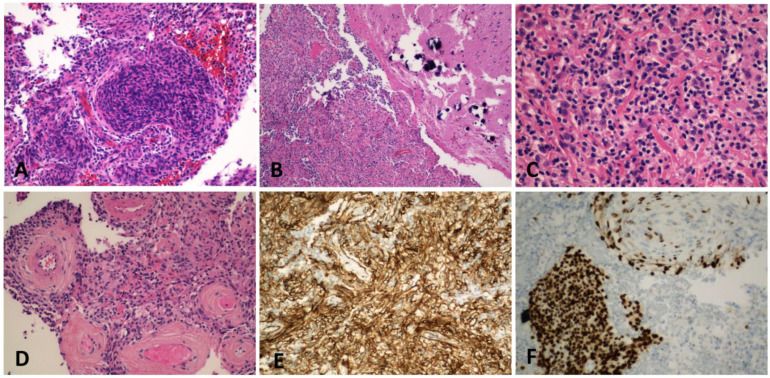
Hematoxylin and eosin staining showing medium-sized meningothelial cells (**A**, 200×); calcifications (**B**, 100×); lymphohistiocytic infiltrate with collagen-rich stroma (**C**, 400×); and thick hyalinized vessels (**D**, 200×); immunohistochemical staining demonstrating lesional cells with strong SSTR2a positivity (**E**, 200×) and focal PR positivity (**F**, 200×).

**Figure 3:**
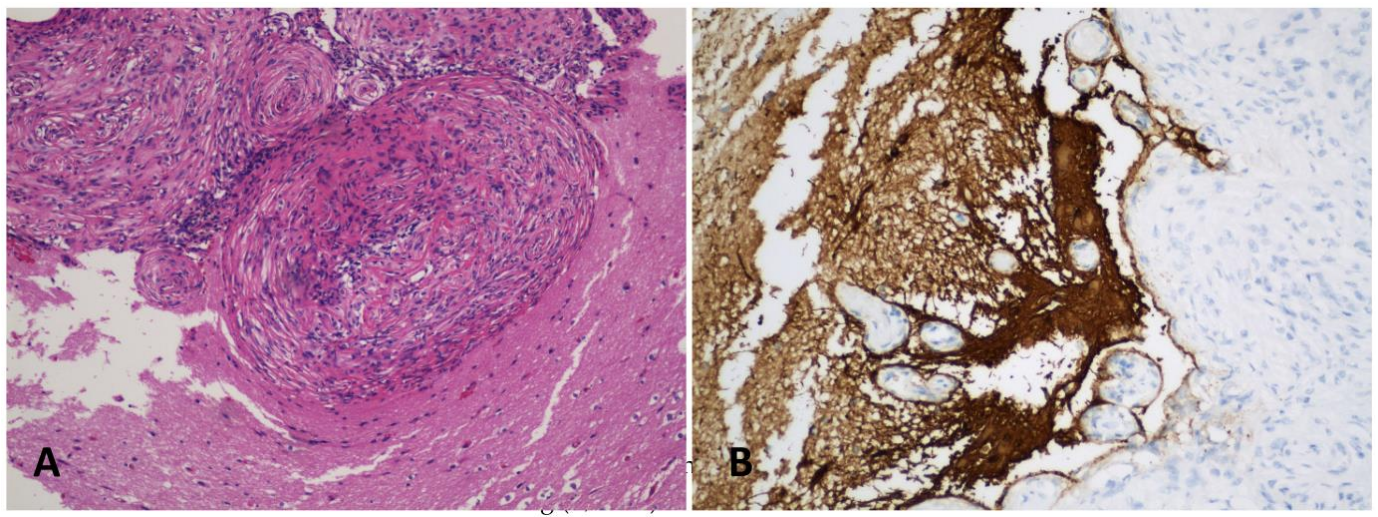
The presence of brain invasion illustrated by hematoxylin and eosin staining (**A**, 100×) and GFAP immunostaining (**B**, 200×).

**Figure 4:**
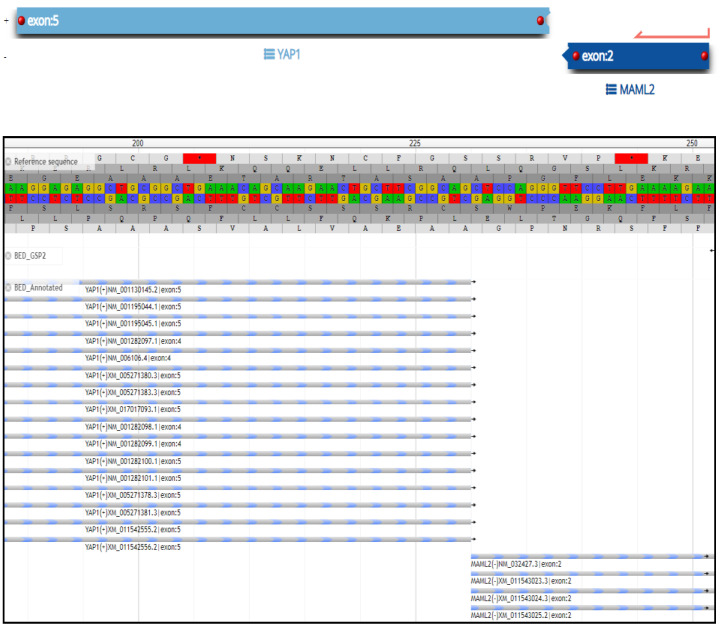
NGS assay of the diagnostic tissue specimen showing the breakpoint region and sequence confirming the YAP1-MAML2 fusion. * it indicates the presence of termination codon.

A 20-year-old male without a medical history had an episode of seizure and was brought into the emergency room. MRI revealed a 1.4 × 1.1 cm lesion with heterogeneous enhancement. The enhancing lesion was located within the right medial temporal lobe inferior and lateral to the right hippocampus. It extended into the cortex without hippocampal or extra-axial involvement (Figure 1). He underwent craniotomy and surgical excision of the lesion. Microscopically, the lesion consisted of medium-sized and spindle-shaped meningothelial cells with moderate nuclear pleomorphism (Figure 2A). Calcifications and collagen-rich stroma were observed (Figure 2B). Significant infiltration of lymphocytes and histocytes was noted (Figure 2C), and prominent vessels were also found associated with the meningothelial cells (Figure 2D). Immunohistochemistry staining showed that the meningothelial cells were strongly positive for somatostatin receptor 2a (SSTR2a) (Figure 2E) and E-cadherin and focally positive for progesterone receptor (PR) (Figure 2F). But, they were negative for epithelial membrane antigen (EMA) and D2-40. The neoplasm exhibited retained ATRX staining and a wild-type p53 expression pattern. EBV in situ was negative. The Ki-67 proliferative index was approximately 3–4%. Notably, GFAP immunostaining demonstrated the presence of focal brain invasion (Figure 3). Targeted RNA fusion NGS analysis evidenced the presence of an in-frame fusion between exon 5 of YAP1 and exon 2 of MAML2 (Figure 4). A further investigation ruled out hematolymphoid neoplasms. The patient was diagnosed with atypical intraparenchymal meningioma with YAP1-MAML2 fusion, WHO grade II.

## 3. Discussion

YAP1 acts as a transcriptional co-activator and plays a significant role in normal tissue development and homeostasis [14,15,16]. Recent studies have indicated that YAP1 activation is frequently associated with the loss of function of the potent tumor suppressor *NF2*/Merlin, which drives tumor growth, invasion, and resistance to apoptosis in various tumors, including meningioma [13,17]. Since YAP1 is negatively regulated by Merlin via the inhibition of its nuclear translocation and transcriptional activity, it is often considered an oncoprotein [10,17]. More recently, YAP1 fusions have been reported in a subset of pediatric meningiomas [6,11]. Further research has shown that the YAP1 fusion *NF2* wild-type meningiomas exhibit high YAP1 activity and express a similar gene profile as *NF2* mutant meningiomas. Gene expression-based clustering analyses of YAP1 point mutations have revealed that YAP1 fusion meningiomas resemble low-grade *NF2* mutant meningiomas based on the up-regulated genes, whereas, based on the down-regulated genes, YAP1 fusion meningiomas cluster with high-grade *NF2* mutant meningiomas [6,13]. Nonetheless, as more cases of YAP1 fusion meningiomas are identified and analyzed, it becomes evident that certain subtypes, such as YAP1-FAM118B fusion, exhibit distinct biological characteristics from the *NF2* mutant [18]. This suggests that YAP1 fusion meningiomas represent a spectrum of complex molecular and histopathological profiles.

The case presented here involves a young adult male with atypical intraparenchymal meningioma harboring YAP1-MAML2 fusion. MRI revealed the tumor located in the right temporal lobe without dural attachment. Microscopically, it was composed of numerous spindle cells with moderate nuclear pleomorphism, dense lymphohistiocytic infiltrate, collagen-rich stroma, and calcifications. In addition, it displayed brain invasion, a criterion for the diagnosis of atypical meningioma, WHO grade II. Non-dural-based intraparenchymal meningiomas are rare, and their exact etiology remains unclear. They are believed to arise from the arachnoid cells and often present with overlapping characteristics with other intracranial tumors and pseudo-tumors, such as vascular malformation and glioma [19]. Distinguishing them based on radiological and morphological findings can be challenging. Immunohistochemistry for meningothelial cells plays a crucial role in accurate diagnosis. Interestingly, a rhabdoid cell feature observed in the previously reported pediatric meningiomas with YAP1-MAML2 fusion was absent in this young adult patient [11].

The YAP1 fusion protein binds to the TEA domain (TEAD) and is most likely to exert resistance to Hippo pathway inhibition, resulting in its hyperactivity. Consequently, hyperactive YAP1 promotes tumor cell proliferation and invasion and plays an oncogenic role in meningioma tumorigenesis. Recent advancement has been made in the search for novel therapeutic approaches to treat meningiomas. Studies have demonstrated that blocking the interaction between YAP1 and TEAD or targeting TEAD auto-palmitoylation can effectively inhibit tumor formation and suppress tumor growth in the YAP1 fusion/*NF2* mutant meningioma and schwannoma [15,20,21]. However, the number of reported YAP1 fusion meningioma cases remains limited. Considering that young patients with meningioma often face a risk of tumor predisposition syndrome, it is highly recommended to perform molecular and genetic testing on tumor tissues for this group of patients. Accumulating a comprehensive database will significantly improve our understanding of the pathological mechanisms of meningioma in order to enable us to optimize the therapeutic approaches.

## 4. Conclusions

The case presented here contributes to the existing database of YAP1-MAML2 meningioma among AYA patients. Given the increased susceptibility to tumor predisposition syndrome within this patient group, comprehensive molecular and genetic analyses on the tumor tissues are strongly suggested. It not only provides additional evidence for diagnosis, but also advances our knowledge of the underlying molecular mechanisms that drives the formation of meningioma.

## Data Availability

Not applicable.

## References

[1] Ostrom Q.T., Gittleman H., Xu J., Kromer C., Wolinsky Y., Kruchko C., Barnholtz-Sloan J.S. (2016). CBTRUS Statistical Report: Primary Brain and Other Central Nervous System Tumors Diagnosed in the United States in 2009–2013. Neuro Oncol..

[2] Ostrom Q.T., Gittleman H., Truitt G., Boscia A., Kruchko C., Barnholtz-Sloan J.S. (2018). CBTRUS Statistical Report: Primary Brain and Other Central Nervous System Tumors Diagnosed in the United States in 2011–2015. Neuro Oncol..

[3] Lin D.D., Lin J.L., Deng X.Y., Li W., Li D.D., Yin B., Lin J., Zhang N., Sheng H.S. (2019). Trends in intracranial meningioma incidence in the United States, 2004–2015. Cancer Med..

[4] Ostrom Q.T., Gittleman H., de Blank P.M., Finlay J.L., Gurney J.G., McKean-Cowdin R., Stearns D.S., Wolff J.E., Liu M., Wolinsky Y. (2016). American Brain Tumor Association Adolescent and Young Adult Primary Brain and Central Nervous System Tumors Diagnosed in the United States in 2008–2012. Neuro Oncol..

[5] Perry A., Giannini C., Raghavan R., Scheithauer B.W., Banerjee R., Margraf L., Bowers D.C., Lytle R.A., Newsham I.F., Gutmann D.H. (2001). Aggressive phenotypic and genotypic features in pediatric and NF2-associated meningiomas: A clinicopathologic study of 53 cases. J. Neuropathol. Exp. Neurol..

[6] Sievers P., Chiang J., Schrimpf D., Stichel D., Paramasivam N., Sill M., Gayden T., Casalini B., Reuss D.E., Dalton J. (2020). YAP1-fusions in pediatric NF2-wildtype meningioma. Acta Neuropathol..

[7] Goutagny S., Bah A.B., Henin D., Parfait B., Grayeli A.B., Sterkers O., Kalamarides M. (2012). Long-term follow-up of 287 meningiomas in neurofibromatosis type 2 patients: Clinical, radiological, and molecular features. Neuro Oncol..

[8] Bachir S., Shah S., Shapiro S., Koehler A., Mahammedi A., Samy R.N., Zuccarello M., Schorry E., Sengupta S. (2021). Neurofibromatosis Type 2 (NF2) and the Implications for Vestibular Schwannoma and Meningioma Pathogenesis. Int. J. Mol. Sci..

[9] Plotkin S.R., Messiaen L., Legius E., Pancza P., Avery R.A., Blakeley J.O., Babovic-Vuksanovic D., Ferner R., Fisher M.J., Friedman J.M. (2022). Updated diagnostic criteria and nomenclature for neurofibromatosis type 2 and schwannomatosis: An international consensus recommendation. Genet. Med..

[10] Zhang N., Bai H., David K.K., Dong J., Zheng Y., Cai J., Giovannini M., Liu P., Anders R.A., Pan D. (2010). The Merlin/NF2 tumor suppressor functions through the YAP oncoprotein to regulate tissue homeostasis in mammals. Dev. Cell.

[11] Zheng X., Guo S., Liu D., Chu J., Li Y., Wang X., Zhang X., Song C., Huang Q. (2022). Pediatric meningioma with a Novel MAML2-YAP1 fusion variant: A case report and literature review. BMC Pediatr..

[12] Esposito S., Marucci G., Antonelli M., Miele E., Modena P., Giagnacovo M., Massimino M., Biassoni V., Taddei M., Schiariti M.P. (2022). Interhemispheric Pediatric Meningioma, YAP1 Fusion-Positive. Diagnostics.

[13] Szulzewsky F., Arora S., Arakaki A.K.S., Sievers P., Almiron Bonnin D.A., Paddison P.J., Sahm F., Cimino P.J., Gujral T.S., Holland E.C. (2022). Both YAP1-MAML2 and constitutively active YAP1 drive the formation of tumors that resemble NF2 mutant meningiomas in mice. Genes Dev..

[14] Yagi R., Chen L.F., Shigesada K., Murakami Y., Ito Y. (1999). A WW domain-containing yes-associated protein (YAP) is a novel transcriptional co-activator. EMBO J..

[15] Szulzewsky F., Holland E.C., Vasioukhin V. (2021). YAP1 and its fusion proteins in cancer initiation, progression and therapeutic resistance. Dev. Biol..

[16] Huang J., Wu S., Barrera J., Matthews K., Pan D. (2005). The Hippo signaling pathway coordinately regulates cell proliferation and apoptosis by inactivating Yorkie, the Drosophila Homolog of YAP. Cell.

[17] Cooper J., Giancotti F.G. (2014). Molecular insights into NF2/Merlin tumor suppressor function. FEBS Lett..

[18] Schieffer K.M., Agarwal V., LaHaye S., Miller K.E., Koboldt D.C., Lichtenberg T., Leraas K., Brennan P., Kelly B.J., Crist E. (2021). YAP1-FAM118B Fusion Defines a Rare Subset of Childhood and Young Adulthood Meningiomas. Am. J. Surg. Pathol..

[19] Jadik S., Stan A.C., Dietrich U., Pietila T.A., Elsharkawy A.E. (2014). Intraparenchymal meningioma mimicking cavernous malformation: A case report and review of the literature. J. Med. Case Rep..

[20] Laraba L., Hillson L., de Guibert J.G., Hewitt A., Jaques M.R., Tang T.T., Post L., Ercolano E., Rai G., Yang S.M. (2023). Inhibition of YAP/TAZ-driven TEAD activity prevents growth of NF2-null schwannoma and meningioma. Brain.

[21] Szulzewsky F., Arora S., Hoellerbauer P., King C., Nathan E., Chan M., Cimino P.J., Ozawa T., Kawauchi D., Pajtler K.W. (2020). Comparison of tumor-associated YAP1 fusions identifies a recurrent set of functions critical for oncogenesis. Genes Dev..

